# Study of glucose uptake activity of *Helicteres isora* Linn. fruits in L-6 cell lines

**DOI:** 10.4103/0973-3930.57349

**Published:** 2009

**Authors:** R. N. Gupta, Anil Pareek, Manish Suthar, Garvendra S. Rathore, Pawan K. Basniwal, Deepti Jain

**Affiliations:** Department of Pharmaceutical Sciences, B.I.T., Mesra, Ranchi- 835 215, Jharkhand, India; 1 L M College of Science and Technology (Pharmacy wing) Jodhpur- 342 003, India; 2 L B S College of Pharmacy, Udai Marg, Tilak Nagar, Jaipur- 302 004, Rajasthan, India; 3 School of Pharmaceutical Sciences, Rajiv Gandhi Technological University, Bhopal, MP, India

**Keywords:** Diabetes, glucose uptake, *Helicteres isora*, skeletal muscles

## Abstract

The effect of hot water extract of fruits of *Helicteres isora* on glucose uptake was studied in rodent skeletal muscle cells (L-6 cells) involved in glucose utilization. *H. isora* is an antidiabetic medicinal plant being used in Indian traditional medicine. Hot water extracts were analysed for glucose uptake activity and found to be significantly active at 200 μg/ml dose comparable with insulin and metformin. Elevation of glucose uptake by *H. isora* in association with glucose transport supported the upregulation of glucose uptake. It was concluded that hot water extract of *H. isora* activate glucose uptake in L-6 cell line of mouse skeletal muscles.

## Introduction

It is well known that the incidence of diabetes mellitus is high all over the world, especially in Asia. Different types of oral hypoglycaemic agents such as biguanides and sulfonylureas available along with insulin for the treatment of diabetes mellitus,[1] have side effects associated with their usage.[2,3] There is a growing interest in herbal remedies because of their efficacy, minimal side effects and relatively low costs. Herbal drugs or their extracts are prescribed widely, even when their biological active compounds are unknown. Even the World Health Organization (WHO) approves the use of plant drugs for different diseases, including diabetes mellitus.[4,5]

Skeletal muscle is the main tissue involved in insulin-induced stimulation of glucose uptake.[6–8] Insulin increases glucose uptake in skeletal muscle by increasing functional glucose transport molecules in the plasma membrane. Glucose transport in skeletal muscle can also be stimulated by contractile activity. The maximal effects of insulin and contractile activity on glucose transport are additive.[9]

In skeletal muscle, both insulin and contractile activity stimulate translocation of glucose transporter GLUT-4 protein from an intracellular membrane pool to the plasma membrane.[9] Resistance to this stimulatory effect of insulin is a major pathological feature of diabetes.[10]

A relatively little-studied response to adrenoceptor activation is facilitation of glucose uptake. Adrenoceptors are classified into three main subtypes: α_1_-, α_2_-, and β-adrenoceptors. β-Adrenoceptor stimulation increases glucose uptake in rodent skeletal muscle[11–14] and brown adipose tissue. This effect is mediated primarily by the β_3_-adrenoceptor in brown adipose tissue,[15,16] it is also mediated by β_2_-adrenoceptors in skeletal muscle cells.[14]

L6 cells represent a good model for glucose uptake because they have been used extensively to elucidate the mechanisms of glucose uptake in muscle, have an intact insulin signaling pathway, and express the insulin-sensitive GLUT4.

*Helicteres isora* L. is a large shrub of the family Sterculiaceae, which occurs, often gregariously, throughout India, from Jamuna eastwards to Bihar and Bengal and southwards in central, western and southern India and Andaman islands. It is one of the best known shrub mentioned in the articles of the Hindu Materia Medica and used as one of the Jamu medicines.[17] It is one of the Jamu raw materials used in traditional folk medicine in Indonesia; it is called “Buah Kayu Ules or Ulet-Ulet” on Java island and is used for the treatment of gastrospasm and as an anthelmintic for tapeworm in Indonesia, and as antispasmodic, antipyretic, antidiarrhoic and antidysenteric in Saudi Arabia[18] and as a tonic compound after childbirth in the Malay Islands.[19]

Timbers of this plant are used as anthelmintic and anti-colic, while fruits are used as anti-colic, anticonvulsant, and in abdominalgia.[20] In addition, Hattori and co-workers reported an inhibitory activity of the water extract of fruits of *H. isora* against reverse transcriptase from avian myeloblastosis virus (AMVRT)[21] and anti-human immunodeficiency virus-type-1 (anti-HIV-1) activity.[22] Traditionally, the root juice is claimed to be useful in diabetes, emphysema, and a favourite cure for snakebite.[23,24] From the roots, betulic acid, daucosterol, sitosterols, isorin[25] can be isolated. Cucurbitacin B and isocucurbitacin B were isolated and reported to possess cytotoxic activity.[26]

Although the hypoglycemic effect of *H. isora* extract has been reported, the exact mechanism of this effect has yet to be elucidated. Therefore, we evaluated the effect of hot water extract of *H. isora* fruits on glucose uptake through glucose transports in skeletal muscle cells (L-6 cell line).

## Materials and Methods

### Plant material

The fruits of *H. isora* (L.) were collected from the local market of Ootacamund, in June 2006 and authenticated by Director, Survey of medicinal plants and collection unit, Ootacamund, Tamilnadu, India.

### Chemicals

Fetal bovine serum was purchased from Invitrogen (Carlsbad, CA, USA). Dulbecco's modified Eagle's medium (DMEM) and other culture products were purchased from GIBCO BRL (San Diego, CA, USA). TPVG solution, Bovine serum albumin (BSA), Insulin, Metformin, Glucose kit was obtained from Randox, Dibasic sodium hydrogen phosphate, sodium bicarbonate, magnesium chloride, calcium chloride, potassium chloride, sodium chloride were from Ranbaxy Laboratories Ltd., Mohali and SD Fine Chem., Mumbai, India. All chemicals and solvents used were of analytical grade.

### Plant extract

The collected fruits were shade dried, coarsely powdered and extracted with hot water by maceration process. The extract was filtered and concentrated in vacuum and kept in a vacuum desiccator for complete removal of solvent. Hot water extract of fruits of *H. isora* was obtained with an yield of 1.8%.

### *In vitro* glucose uptake activity

#### Preparation of cell culture

Monolayer of L-6 cells was maintained at sub confluent conditions in growth media containing DMEM with 4.5 g/l glucose, 100 IU/ml penicillin, 100 μg/ml streptomycin, and 10% fetal bovine serum. Cells were maintained in a humidified 37°C incubator with ambient oxygen and 5% CO_2_. Cells were maintained in continuous passage by trypsinization of sub confluent cultures using TPVG solution.

#### Glucose uptake assay

Cells were cultured on 6 well plates and incubated for 48 h at 37°C in a CO_2_ incubator. When semi confluent monolayer was formed, the culture was renewed with serum free DMEM containing 0.2% BSA and incubated for 18 h at 37°C in the CO_2_ incubator. After 18 h, the media was discarded and cells were washed with KRP buffer once. The cells were treated with Insulin, standard drug and plant extract and added glucose (1M) and incubated for half an hour. The supernatant was collected for glucose estimation and glucose uptake was terminated by washing the cells thrice with 1 ml ice-cold KRP buffer. Cells were subsequently lysed by freezing and thawing thrice. Cell lysate was collected for glucose estimation.

Glucose uptake was calculated as the difference between the initial and final glucose content in the incubated medium by GOD-POD method as follows:

Mix 10 μl of sample and 1 ml of reagent, incubate for 25 min at 15-25°C or 10 min at 37°C. Measure the absorbance of the standard (A_standard)_ and the sample (A_sample_) against the reagent blank within 60 min, the time interval from sample addition to read time must be exactly the same for standard/control and sample.

$\begin{array}{ll} \text{Glucose concentration} & \text{mmol} \end{array}/1=\frac{{\text{A}}_{\text{sample}}}{{\text{A}}_{\text{standard}}}\times 55.5$

$\text{mg/dl}=\frac{{\text{A}}_{\text{sample}}}{{\text{A}}_{\text{standard}}}\times 100$

Six groups containing five wells of plate (*n* = 5) each were taken as given in Table 1.

**Table 1:** Incubation medium used for glucose uptake assay in L-6 cell line

S. No	Incubation medium
Group 1.	900 μl of KRP buffer and 100 μl of glucose solution (1M) (control group)
Group 2	800 μl of KRP buffer, 100 μl of Insulin (10 IU/ml) and 100 μl of glucose solution (1M).
Group 3	800 μl of KRP buffer, 100 μl of metformin (1mg/ml) and 100 μl of glucose solution (1M).
Group 4	700 μl of KRP buffer, 100 μl of Insulin (10 IU/ml),100 μl of metformin (1mg/ml) and 100 μl of glucose solution (1M).
Group 5	800 μl of KRP buffer, 100 μl of plant extract (2mg/ml) and 100 μl of glucose solution (1M).
Group 6	700 μl of KRP buffer, 100 μl of Insulin (10 IU/ml), 100 μl of plant extract (2 mg/ml) and 100 μl of glucose solution (1M).

## Results and Discussion

**Figure 1:**
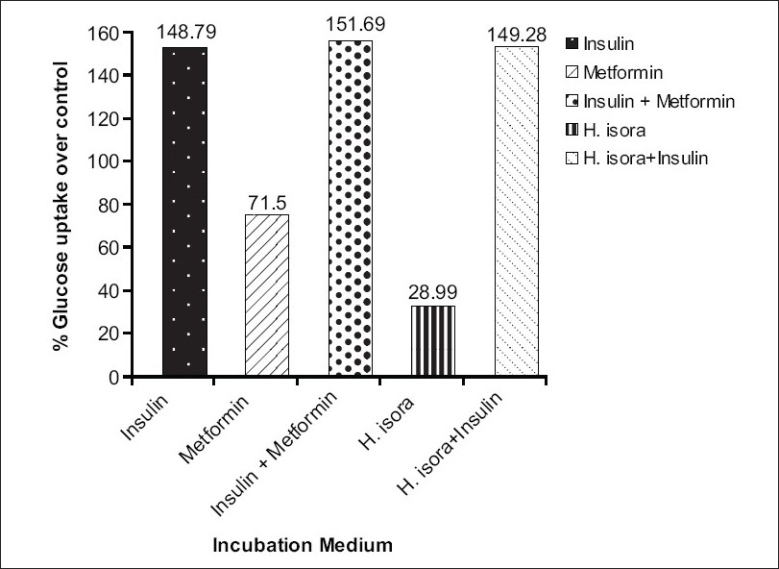
Effect of hot water extract of fruits of *Helicteres isora* on glucose uptake in L-6 cell line

Glucose utilization in L-6 cell lines was studied *in vitro*. The results are given in Table 2 and Figure 1. The given results show that hot water extract of fruit of *H. isora* enhance the glucose uptake by 28.99 % over control at 200 μg/ml dose. Results were compared with insulin (injectable anti diabetic) and metformin (oral anti diabetic), which were used as the standard anti diabetic drugs. Insulin (1IU/ml) and metformin (100 μg/ml) enhance the glucose uptake by 148.79 % and 71.50% over control. Extract was also tested with insulin to confirm any synergistic effect, but results indicate that extract does not have any synergistic effect with insulin. Extract and insulin enhance the glucose uptake in L-6 cells by 149.28% over control when used in combination.

**Table 2:** Effect of hot water extract of fruits of Helicteres isora on glucose uptake in L-6 cell line.

S.No	Incubation medium	% Glucose uptake over control
Group 1.	Insulin (1 IU/ml)	148.79
Group 2	Metformin (100 μg/ml)	71.50
Group 3	Insulin (1 IU/ml) + metformin (100 μg/ml)	151.69
Group 4	Helicteres isora extract (200 μg/ml)	28.99
Group 5	Helicteres isora extract (200 μg/ml) + insulin (1 IU/ml)	149.28

Skeletal muscle is the primary site responsible for postprandial glucose use. Furthermore, it is the most abundant tissue in the whole body, and thus, proper function of skeletal tissue is important to maintain normal blood glucose level.[27] Defects in insulin stimulated skeletal muscle glucose uptake are common pathological states in non-insulin-dependent diabetes mellitus (NIDDM).[28] GLUT4 is the major glucose transporter expressed in insulin responsive tissue such as skeletal muscle and adipose tissue, where they respond to an acute insulin challenge by translocating GLUT4 rapidly from an intracellular membrane storage site to the plasma membrane.[29]

The results obtained in the present study clearly demonstrate that the *H. isora* hot water extract enhances glucose uptake under *in vitro* conditions. This may be due to its effect on the number of receptors located in the skeletal muscle cell line.
